# Supplementary material for: Content-rich biological network constructed by mining PubMed abstracts
Source: BMC Bioinformatics. 2004 Oct 8;5:147. doi: 10.1186/1471-2105-5-147 (PMC528731; doi:10.1186/1471-2105-5-147)
Supplement: Additional File 2 — The original results of the above study (non-essential files are deleted to keep the file size under the limit set by BMC bioinformatics). [file 1471-2105-5-147-S2.bz2 › chilibotAdditionalFile2/dip05/379ID9927421E9792/html/NGFR_NTRK3.html]

 


 **NGFR** and **NTRK3** 
  
Found 76 abstracts in PubMed, retrieved 05.  
 

 What does Google say? 
 PDF only 
| .edu only 

---

**Interactive relationship** (e.g. stimulation, inhibition, etc)

**Non-interactive relationship** (e.g. studied together, co-existance, homology, etc.)

- Mouse PTPRO mRNA was observed in neuron populations expressing TrkA, the high affinity nerve growth factor receptor  [ **NGFR** ] , or TrkC  [ **NTRK3** ] , the neurotrophin 3 receptor  [ **NTRK3** ] , and immunoreactive mPTPRO and TrkC  [ **NTRK3** ]  colocalized in large dorsal root ganglia proprioceptive neurons.  Ref: 12532410 J Comp Neurol, 2003
- Although neurotrophins did not regulate p75NTR  [ **NGFR** ]  processing, the alpha and gamma secretase mediated cleavage of p75 is modulated by receptor tyrosine kinases Trks TrkA and TrkB but not TrkC  [ **NTRK3** ] .  Ref: 12843241 J Neurosci, 2003
- Our results show that myelin formation is inhibited in the absence of functional p75NTR  [ **NGFR** ]  and enhanced by blocking TrkC  [ **NTRK3** ]  activity.  Ref: 12424382 Science, 2002
- Thus p75NTR  [ **NGFR** ]  and TrkC  [ **NTRK3** ]  receptors have opposite effects on myelination.  Ref: 12424382 Science, 2002
